# Zebra bodies recognition by artificial intelligence (ZEBRA): a computational tool for Fabry nephropathy

**DOI:** 10.1038/s41598-026-35466-w

**Published:** 2026-01-12

**Authors:** Giorgio Cazzaniga, Maurizio Carbone, Raffaella Barretta, Gabriele Casati, Simona Vatrano, Giovanni Gambaro, Gisella Vischini, Irene Capelli, Renzo Mignani, Gianandrea Pasquinelli, Federico Pieruzzi, Leonardo Caroti, Egrina Dervishi, Marco Allinovi, Luca Novelli, Antonio Pisani, Albino Eccher, Fabio Pagni, Vincenzo L’Imperio

**Affiliations:** 1 Pathology, Fondazione IRCCS San Gerardo Dei Tintori, Monza, Italy; 2 School of Medicine and Surgery, University of Milano-Bicocca, Milan, Italy; 3 Pathology Unit, Gravina Hospital Caltagirone ASP, Catania, Italy; 4 Division of Nephrology, Azienda Ospedaliera Universitaria Integrata Verona, and Department of Medicine, University of Verona, Verona, Italy; 5 Nephrology, Dialysis and Renal Transplant Unit, IRCCS Azienda Ospedaliero-Universitaria di Bologna, Bologna, Italy; 6 Department of Medical and Surgical Sciences (DIMEC), Alma Mater Studiorum University of Bologna, Bologna, Italy; 7 Department of Nephrology, Infermi Hospital, Rimini, Italy; 8 Pathology Unit, IRCCS Azienda Ospedaliero-Universitaria di Bologna, Bologna, Italy; 9 Clinical Nephrology, Fondazione IRCCS San Gerardo Dei Tintori, Monza, Italy; 10 Nephrology, Dialysis and Transplantation Unit, Careggi University Hospital, Florence, Italy; 11 Institute of Histopathology and Molecular Diagnosis, Careggi University Hospital, Florence, Italy; 12 Nephrology, University Federico II, Naples, Italy; 13 Department of Medical and Surgical Sciences for Children and Adults, University of Modena and Reggio Emilia, University Hospital of Modena, Modena, Italy

**Keywords:** Digital pathology, Fabry nephropathy, Renal biopsy, Artificial intelligence, Computational pathology, Computational biology and bioinformatics, Diseases, Nephrology

## Abstract

Fabry disease (FD) is a rare lysosomal storage disorder caused by mutations in the GLA gene, resulting in globotriaosylceramide accumulation. Kidney involvement (Fabry nephropathy) significantly contributes to morbidity and mortality. Diagnosis can be difficult, especially in females or late-onset variants. Renal biopsy remains essential, but interpretation requires expert pathologists. Digital pathology and artificial intelligence (AI) offer promising solutions to support diagnosis. The study analyzed Whole-slide images from renal biopsies of Fabry nephropathy patients to develop and validate a “foamy podocytes” screening AI tool. Two computational tasks were performed: glomerular-level classification, and podocyte-level segmentation. Performance was evaluated using standard metrics. A novel ZEBRA score (fpA/tgA%) was developed to quantify disease burden, and correlations with histological scores and clinical parameters were assessed. EfficientNetB2 achieved the highest classification accuracy (79%) in identifying foamy podocytes. SegFormerB4 had the best segmentation performance (Dice = 0.46, IoU = 0.37). The ZEBRA score effectively distinguished Fabry nephropathy from controls (*p* < 0.001) and showed good correlation with manual scoring (rs = 0.66–0.71). The AI-assisted ZEBRA pipeline highlights high-risk Fabry nephropathy features to support nephropathologists as a screening tool.

**Supplementary Information:**

The online version contains supplementary material available at 10.1038/s41598-026-35466-w.

## Introduction

Fabry disease (FD) is a multisystem lysosomal storage disorder caused by mutations in the GLA gene on the X chromosome, leading to a deficiency of α-galactosidase A (AGAL). This enzymatic defect results in the accumulation of globotriaosylceramide (Gb3) in various organs, including the heart, nervous system, skin, and kidneys^1^. Kidney involvement (Fabry nephropathy, FN) is a major contributor to morbidity and mortality^2,3^ with progressive proteinuria that may lead to end-stage kidney disease (ESKD) if not promptly recognized and treated^4,5^. However, FN is often clinically silent in early stages, especially in late-onset or atypical variants and in female patients due to X-chromosome inactivation (lyonization), making early diagnosis challenging^6^. In this context, renal biopsy remains crucial for detecting early Gb3 deposits via light and electron microscopy, even before other systemic signs emerge^7–9^. Histological evaluation also supports treatment decisions^8^, monitoring response^9–11^ and predicting disease progression^9,12,13^. Currently, diagnosis relies on the expertise of pathologists in identifying podocyte cytoplasmic vacuolization—FN’s hallmark—which may be subtle or focal, particularly in atypical or female cases^9,14^, representing a potential challenge for non experienced nephropathologists. Although vacuolated podocytes often provide the only morphological hint of FN on biopsy, their presence is not pathognomonic and must be interpreted with caution, as similar appearances can be observed in other lysosomal storage disorders or in drug-induced phospholipidosis^15^. Confirmatory electron microscopy, identifying characteristic lamellated inclusions (e.g., myelin and zebra bodies)^16^, is not always available, increasing the risk of underdiagnosis. As kidney involvement may precede other symptoms^17^, renal biopsy can be the first clue to FD, prompting genetic and biochemical testing. Digital pathology may represent the ideal solution to overcome these challenges, since the conversion of physical slides into whole slide images (WSIs) enable the creation of hub-spoke centers with high expertise in the evaluation of these rare diseases^18,19^, favoring both the preservation of high quality standards and the development of artificial intelligence (AI) and computational tools to assist the pathologists in the evaluation of such cases^20^. Although recent computational nephropathology efforts have successfully applied AI to segment renal tissue (e.g., glomeruli, tubules, interstitium)^21^ and classify glomerular lesions (e.g., sclerosis, hypercellularity)^22^, these models often focus on broad morphological classes and not on rare and disease-specific features such as podocyte vacuolization. Here, a computational pipeline for the automatic detection of vacuolated podocytes is developed and validated as a scalable, automated risk-alert tool that sensitively screens for and quantifies the specific morphological hallmarks of FN.

## Methods

### Cases

A multicenter analysis of formalin-fixed and paraffin-embedded renal biopsy and genetically-confirmed FNs was conducted across different Italian institutions (Table 1), spanning a time period of 15 years (2010–2024). For each case (*n* = 37) and control (*n* = 40), a minimum of one 2–3 μm cut hematoxylin and eosin (H&E) or periodic acid-Schiff (PAS) stained slide was obtained, including both staining types when available. Cases were included if they underwent kidney biopsy according to standard nephrological indications, regardless of whether FD was initially suspected. Indications for biopsy included persistent significant proteinuria (> 0.5–1 g/day), hematuria, unexplained progressive decrease in renal function, or other clinical or laboratory signs suggesting underlying renal disease. Controls were randomly selected from cases with an alternative final diagnosis and included only glomeruli evaluated as devoid of the lesion of interest upon histopathological review; matching by center was applied whenever possible, and matching by time period was ensured by selecting slides from the same year. For all cases, electron microscopy was performed confirming the presence of myelin bodies in FN cases and their absence in controls. The slides were scanned using different whole-slide imaging systems at varying magnifications. Demographic and clinical data were collected, including sex, age, serum creatinine levels (mg/dL), estimated glomerular filtration rate (eGFR) following the CKD-EPI formula (ml/min/1.73 m^2^), proteinuria (g/24 h), GLA mutation type, associated amino acid change, and FD clinical phenotype. Pathological features were evaluated by a renal pathologist (VL) for each biopsy, documenting: the total number of glomeruli, the number of globally sclerotic glomeruli, the number of glomeruli with segmental sclerosis, the mean podocyte vacuolization score based on the International Study Group of FN (ISGFN) scheme^9^, and the estimated percentage of interstitial fibrosis and tubular atrophy (IFTA%). This study complies with the Declaration of Helsinki and was performed according to ethics committee approval (PNRR-MR1-2022–12375735, n. 16681, 03/16/23).

**Table 1:** Technical characteristics of the WSIs included in the dataset, categorized by center, staining method, scanner type, and presence of foamy podocytes. Numerical values in parentheses indicate the number of glomeruli in each category. Spatial resolution of digital images: 0.2208 μm/pixel for nanozoomer S60 (Hamamatsu, Shizuoka, Japan); 0.2506 μm/pixel for MIDI II (3DHISTECH, Budapest, Hungary), 0.25 μm/pixel for KF-PRO-400 (KFBIO, Ningbo, China).

Dataset Split (glomeruli)	Center* (glomeruli)	Cases/Slides	Stains (glomeruli)	Scanners (glomeruli)	Disease (glomeruli)
**Training/** **Validation** (1227)	**DIPLOMAT** (479)	FN(8/15) Controls(14/29)	H&E (243) PAS (236)	MIDI II (135) S60 (344)	FN: 245 Controls: 234
	**Bologna** (679)	FN(16/51) Controls(18/52)	H&E (302) PAS (377)	MIDI II (679)	FN: 406 Controls: 273
	**Naples** (119)	FN(6/6) Controls(-)	H&E (56) PAS (63)	MIDI II (56) S60 (63)	FN: 119
**Test** (487)	**Florence** (487)	FN(7/13) Controls(8/16)	H&E (248) PAS (239)	KF-PRO (305) S60 (182)	FN: 305 Controls: 182

*Cases were collected within a DIPLOMAT trial (Fondazione IRCCS San Gerardo dei Tintori - University of Milano-Bicocca, Monza, Italy; Azienda Sanitaria Provinciale (ASP) Catania, “Gravina” Hospital, Caltagirone, Italy; and Department of Medicine, University of Verona, Verona, Italy) and from Policlinico Sant’Orsola-Malpighi, Bologna; Azienda Ospedaliera Universitaria Careggi, Florence; and University Federico II, Naples.

## Dataset creation and model development

**Fig. 1:**
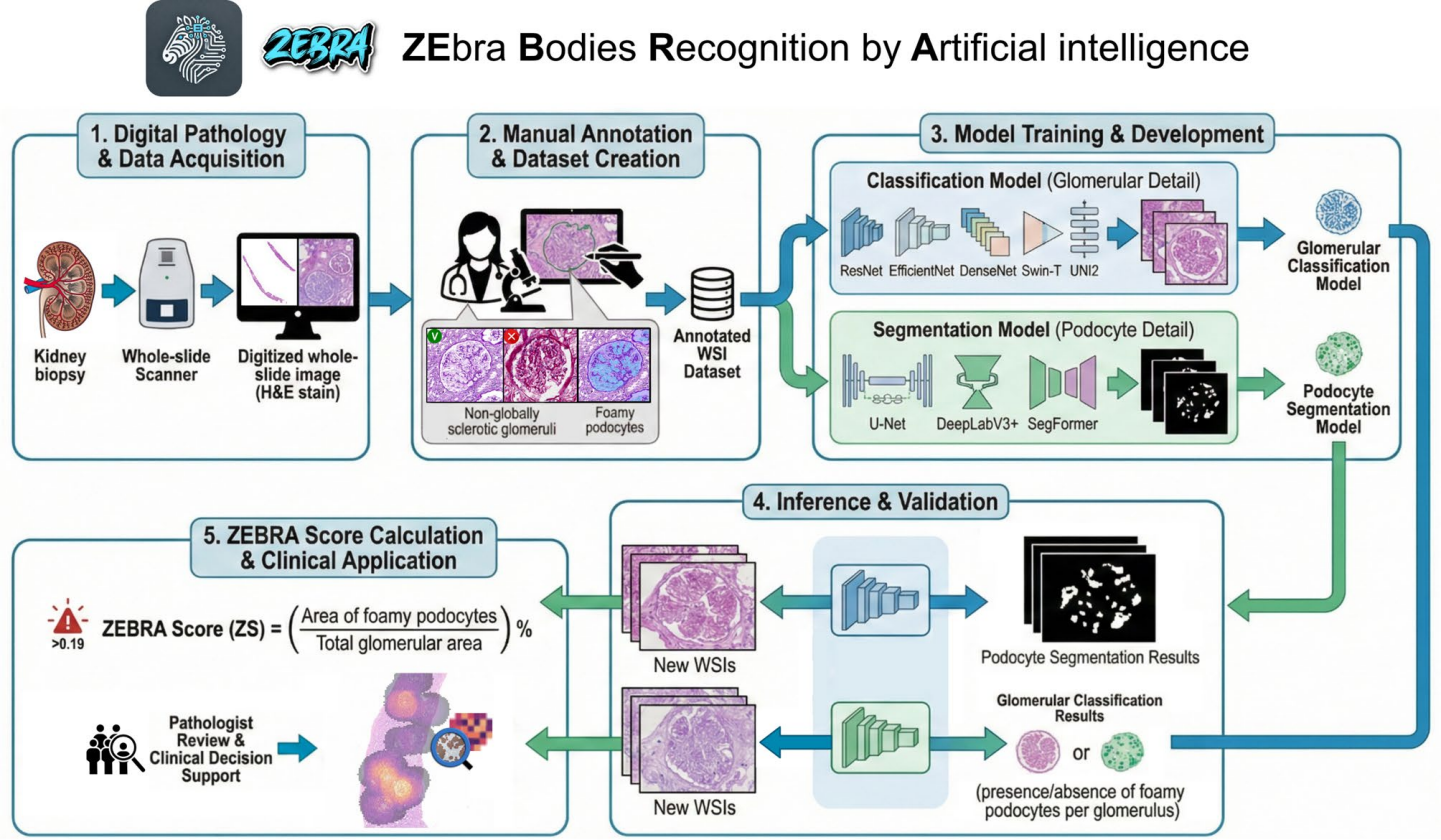
Schematic representation of the computational pathology pipeline for the detection of foamy podocytes. After case selection and subdivision in training/validation and test sets, biopsies were annotated at both glomerular level (A: giving a label of foamy or not foamy, green and red in the figure) and at podocyte level (B: delineating the podocyte with foamy cytoplasm). Based on these annotations, cases were used to build a classification and segmentation model, for A and B annotations respectively. The obtained algorithms were applied on an external test set to evaluate their performances on either the classification of positive/negative glomeruli of the biopsy (glomerular detail) or the outlining of foamy podocytes within glomeruli (podocyte detail). The obtained pipeline (ZEbra Bodies Recognition by Artificial intelligence, ZEBRA) is available as a free QuPath-WSInfer extension (classification system) as and as a segmentation tool freely available as a GitHub repository.

In the initial phase, non-globally-sclerotic glomerular regions and individual foamy podocytes were manually annotated using QuPath v0.5.1^23^ by a pathology resident (MC) on a pathology-dedicated medical monitor (BARCO MDPC-8127, Courtrai, Belgium). The annotations were subsequently reviewed and corrected by an expert nephropathologists (FP) with over 15 years of experience in renal biopsy interpretation. Based on these annotations, 512 × 512 pixel tiles were extracted at a magnification of 0.5 μm/px and categorized according to the presence or absence of foamy podocytes. Two complementary experiments were conducted to automate the detection and analysis of foamy podocytes within glomerular regions, using independently trained models:

- a classification model designed as a computationally lightweight, easily integrable screening tool compatible with existing frameworks (WSInfer and QuPath);
- a separate segmentation model developed for detailed, feature-level quantification to provide explainability and support granular disease assessment (Fig. 1).

We additionally tested the model on a rare case of crystalline podocytopathy, still characterized by a foamy-like appearance, to check whether the model is reliable when stressed with ambiguous cases.

1. Classification experiment (glomerular detail):

A series of classification models were trained to detect the presence/absence of foamy podocytes at the glomerular level. These models included ResNet18, EfficientNetB2, DenseNet121, Swin-T, and a custom architecture that utilized UNI2 (a foundational model developed by the Mahmood Lab at Harvard)^23^. Training was performed using 5-fold cross-validation at the case level, ensuring that all tiles from a single case were consistently assigned to either the training or validation set in each fold, with evaluations performed on an independent test set. The test set did not include any slides from the internal cohort. Each model was trained with and without data augmentation (Supplementary Methods). Performance on an external test set was evaluated at both the tile and case levels, using standard classification metrics: accuracy, precision, recall, F1 score, receiver operating characteristic-area under the curve (AUC-ROC), sensitivity, specificity, positive predictive value (PPV), negative predictive value (NPV). The 95% confidence intervals for metrics were estimated using a bootstrap approach with 1,000 resamples, sampling with replacement from the predictions and recalculating each metric to derive the empirical percentile-based intervals.

2. Segmentation experiment (podocyte detail):

Several segmentation models were employed, including U-Net, DeepLabV3+, and SegFormerB4. The same 5-fold case-level cross-validation scheme was applied, with evaluations performed on an independent test set. Model performance was assessed at both the tile and case levels, using the Dice coefficient and intersection over union (IoU) metrics to measure concordance with manually annotated masks. The Dice coefficient quantifies the overlap between predicted and reference regions relative to their combined size, while IoU measures the overlap relative to the union of the predicted and reference regions. Although calculated differently, these metrics are closely related and provide complementary information on segmentation accuracy. In addition, a separate model was also trained specifically to segment the glomerulus, enabling accurate delineation of the glomerular area and supporting the subsequent quantification of foamy podocyte proportions. For each glomerulus, the proportion of the area occupied by foamy podocytes (fpA) on the total glomerular area (tgA) was calculated (fpA/tgA%) and correlated with the ISGFN scoring system^9^.

### Statistical analysis

Continuous variables were summarized as medians and interquartile ranges (IQR), while categorical variables were expressed as counts and percentages. Differences in continuous and categorical distributions were evaluated using the Mann-Whitney U test and χ² test, respectively, with statistical significance defined as *P* < 0.05. To evaluate the agreement between the continuous values derived from the manual mean podocyte vacuolization score (MPVS, as determined by ISGFN^9^ and the outputs of the automated segmentation models estimating the glomerular area occupied by foamy podocytes, the Spearman correlation coefficient (r_s_) was used. To evaluate the discriminative performance of the ZEBRA score between FN and controls, a receiver operating characteristic (ROC) curve analysis was performed. The AUC was calculated to quantify overall performance. The optimal cutoff value was determined using Youden’s J statistic, and the corresponding sensitivity and specificity were reported to provide guidance for potential clinical interpretation. Linear regression (R^2^) was used to assess the correlation of the MPVS and the computational score with biochemical values and ANCOVA test for statistical significance. All statistical analyses were performed using Excel 2016 (Microsoft) and Python v.3.12 with the libraries pandas v.2.2.3 and scikit-learn v.1.4.2.

## Results

### Cases

The clinicopathological features of the cohort are presented in Table 2. Controls were composed of 12 IgA nephropathy, 6 Minimal change disease and 6 arterionephrosclerosis, 5 minimal histological abnormalities, 4 membranous nephropathy and 4 focal and segmental glomerulosclerosis and 3 miscellaneous (thrombotic microangiopathy, fibrillary glomerulonephritis and proliferative glomerulonephritis with monoclonal immunoglobulin deposits) cases. The distribution of GLA mutations among Fabry patients revealed a predominance of the c.644 A > G and c.1066 C > T variants, with the majority of patients exhibiting the late-onset (LO) phenotype (73%). FN demonstrated higher eGFR (98 ml/min/1.73m^2^ vs. 69 ml/min/1.73m^2^ *p* < 0.001), lower levels of proteinuria (median 0.2 g/day vs. 1.28 g/day, *p* < 0.001) and milder IFTA (*p* = 0.01) compared to controls. Additionally, FNs showed a median podocyte vacuolization score of 1.18 (IQR 0.89–2.05). The dataset comprised 1,075 glomeruli from FN cases and 689 from controls; vacuolized podocytes were identified in 74% (796/1,075) of the FN glomeruli, resulting in a final distribution of 796 positive and 968 negative glomeruli.

**Table 2:** Clinical, demographic, genetic and histological features of the cohort.

	Fabry (*n* = 37)	Controls (*n* = 40)	*p* value
Clinical and laboratory data			
*Sex (F*,* n and %)*	19 (51%)	12 (30%)	-
*Age (median and IQR)*	46 [33–55]	52 [32–63]	-
*Serum Creatinine (mg/dl*,* median and IQR)*	0.83 [0.70–0.98]	1.2 [0.83–1.60]	0.38
*eGFR (ml/min/1.73 m*^*2*^, *median and IQR)*	98 [76–110]	69 [44–95]	**< 0.001**
*Proteinuria (g/die*,* median and IQR)*	0.2 [0.11–0.33]	1.28 [0.36–3.43]	**< 0.001**
*Fabry phenotype (LO*,* n and %)*	27 (73%)	-	-
GLA mutation *(n and %)*			
*c.644 A > G*	15 (41%)	-	-
*c.1066 C > T*	8 (22%)	-	-
*c.1121_1123delAAG*	2 (5%)	-	-
*c.4 C > T*	2 (5%)	-	-
*c.902G > C*	2 (5%)	-	-
Other*	8 (22%)	-	-
Histological data *(median and IQR)*			
*N of glomeruli*	15 [8–22]	11 [9–17]	0.28
*N of globally sclerosed glomeruli*	1 [0–2]	2 [0–3]	0.28
*N of segmentally sclerosed glomeruli*	0	0 [0–1]	0.1
*IFTA (%)*	5 [5–5]	10 [5–20]	**0.01**
*Mean vacuolization podocyte score*	1.18 [0.89–2.05]	-	-
Final diagnosis *(n)*			
*Fabry nephropathy*	37	-	-
*IgA nephropathy*	-	12	-
*Minimal Change Disease*	-	6	-
*Membranous nephropathy*	-	4	-
*Arterionephrosclerosis*	-	6	-
*Minimal histological abnormalities*	-	5	-
*Focal & segmental glomerulosclerosis*	-	4	-
Other**	-	3	-

**1 case for each type of mutation (c.1091_1092del; c.272T > C; c.658 C > T; c.667T > G; c.704 C > G; c.73delG; c.886 A > T; c599_560 del AT).

**1 case each for Thrombotic Microangiopathy, Fibrillary Glomerulonephritis (DNAJB positive) and Proliferative glomerulonephritis with monoclonal immunoglobulin deposits (PGMID).

### Classification task (glomerular detail)

**Fig. 2:**
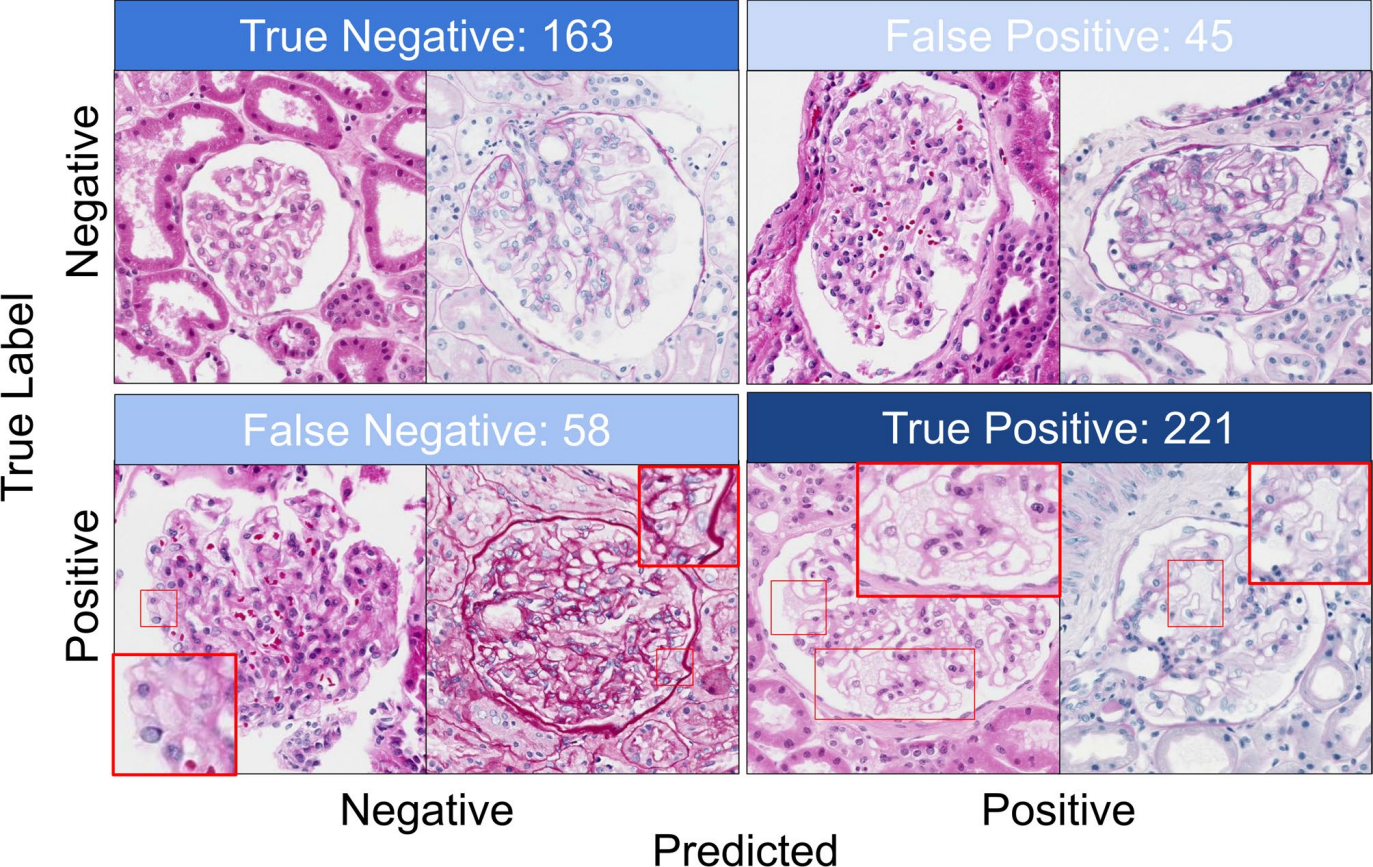
The confusion matrix shows the exact number of correctly classified and misclassified glomeruli for the best model. For illustration, two representative examples are provided for each prediction category (correctly classified and misclassified), with zoomed-in views highlighting the foamy podocytes.

The performance metrics of all models on the training, validation, and test sets are presented in Table 3. The application of augmentation techniques enhanced the generalizability of the models, as evidenced by only a minor reduction in performance across the training, validation, and test sets (Table 3). EfficientNetB2 models achieved the highest performance in glomerular classification at the tile level on the external test set, with an accuracy, precision, recall, F1-score, sensitivity and AUC-ROC of 0.79, a PPV of 0.78, specificity of 0.83, and NPV of 0.74 (Fig. 2). This model successfully identified all FNs as positive at case level, although it misclassified at least one glomerulus in 10 out of 16 controls in the external test set, resulting in a perfect NPV and sensitivity (1), a PPV of 0.57 and a specificity of 0.38 at case level. These findings suggest the potential role of this classifier as a screening tool to detect cases that may benefit from a secondary review by pathologists.

**Table 3:** Classification and segmentation metrics of the different models tested with and without augmentation in training, validation and test sets.

Classification		Train					Validation					Test				
		Acc	Pr	Rc	F1	Auc	Acc	Pr	Rc	F1	Auc	Acc	Pr	Rc	F1	Auc
*ResNet*		93	93	93	93	93	74	74	75	73	75	61 [57–66]	71 [64–78]	55 [53–57]	47 [44–52]	55
	*aug	88	88	88	88	88	77	77	76	78	76	75 [71–79]	76 [72–80]	73 [69–76]	73 [69–77]	73
*EfficientNet*		92	92	92	92	92	75	74	76	74	76	58 [54–63]	58 [48–66]	52 [50–54]	43 [40–47]	52
	*aug	87	87	87	87	87	76	77	78	76	78	79 [75–83]	78 [75–82]	79 [75–82]	79 [75–82]	79
*DenseNet*		93	93	93	93	93	76	75	77	75	77	57 [53–62]	29 [27–31]	50 [50–50]	36 [35–38]	50
	*aug	89	89	89	89	89	78	77	79	77	79	73 [69–77]	75 [72–80]	70 [66–74]	70 [66–74]	70
*Swin-T*		86	86	86	86	86	81	80	82	80	82	64 [60–68]	69 [63–74]	59 [56–62]	55 [51–60]	59
	*aug	80	80	80	80	80	81	81	81	81	81	78 [75–82]	78 [75–82]	79 [75–82]	78 [75–82]	79
*UNI2*		85	85	85	85	85	80	80	80	80	80	66 [62–70]	74 [70–77]	69 [66–73]	65 [61–69]	69
	*aug	79	79	79	79	79	79	79	80	79	80	63 [59–67]	74 [71–77]	68 [64–70]	61 [57–66]	67
*Segmentation*																
		Dice	IoU				Dice	IoU				Dice	IoU			
*Unet*		21	13				18	11				16	12			
	*aug	14	8				14	9				26	22			
*DeepLabV3*		57	41				33	23				24	15			
	*aug	29	18				23	14				24	14			
*SegFormer*		63	49				52	44				46	37			
	*aug	31	20				38	30				25	17			
*Glomeruli* *(SegFormer)*		97 ± 1	92 ± 1				95 ± 1	89 ± 1				94	88			
	*aug	95 ± 1	89 ± 1				94 ± 1	88 ± 1				94	88			

### Segmentation task (podocyte detail)

**Fig. 3:**
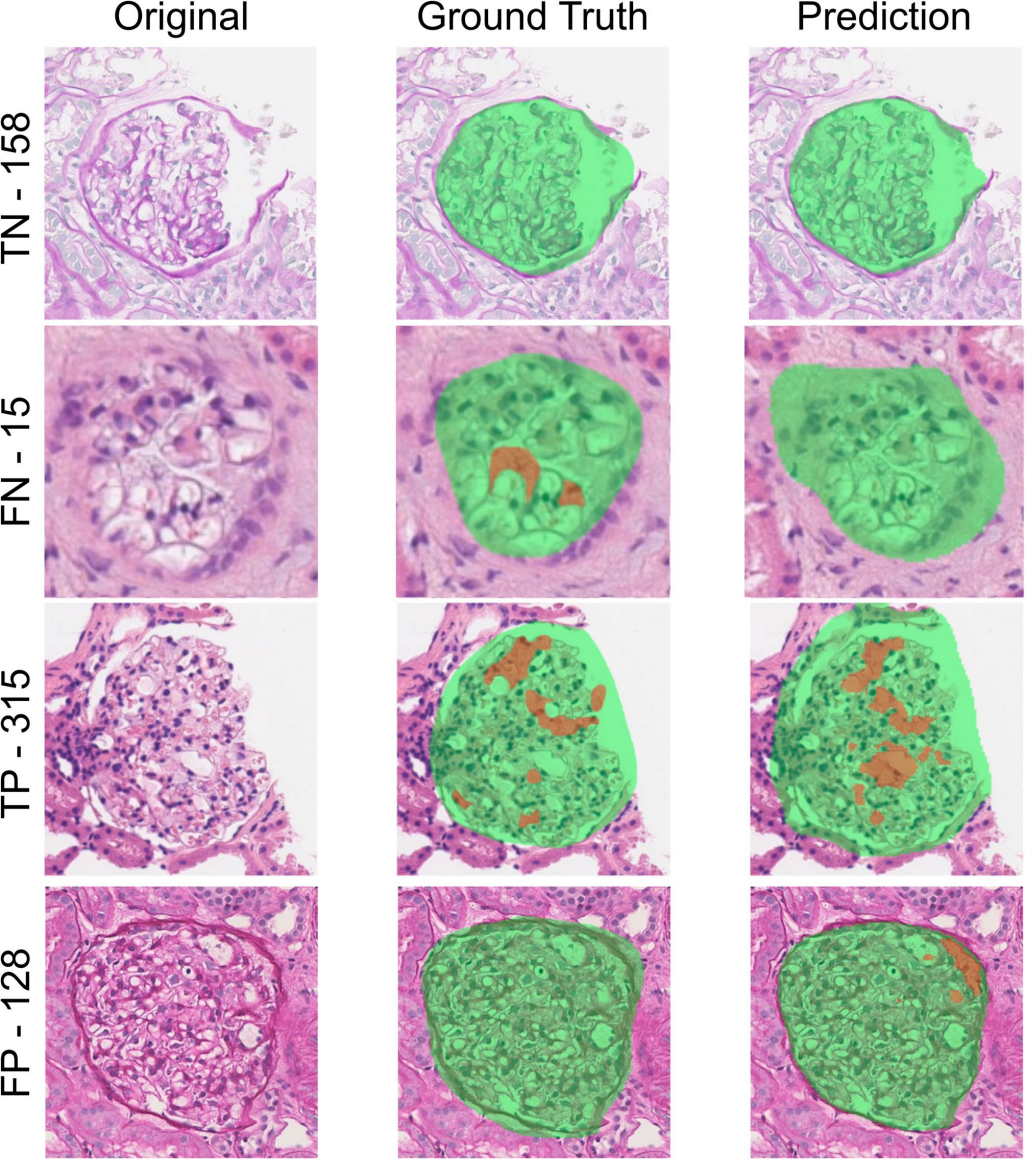
Examples of cases correctly classified or misclassified by the segmentation model. In green, the segmented glomerular area and in orange the vacuolized podocytes are shown. TN=True Negative, FN = False Negative, TP = True Positive, FP = False Positive. The reported values refer only to glomeruli that were correctly segmented by the model, with a sample considered positive when at least one pixel was predicted as a vacuolized podocyte.

The performance metrics of all models on the training, validation, and test sets are presented in the Supplementary Results. Among the different models tested (Table 3), the SegFormer B4 achieved the highest performances in foamy podocyte segmentation (Dice = 0.46 and IoU = 0.37 without augmentation), with a tile-level sensitivity of 0.95 and a PPV of 0.91, where the tile-level assessment specifically refers to detecting the presence or absence of foamy podocytes based on the segmentation output, further stressing the role of this AI tool as a screening assistant (Fig. 3).

To streamline the inference workflow, a custom script was developed to automatically generate region annotations at the appropriate scale to be manually positioned around glomeruli (ZEbra Bodies Recognition by Artificial intelligence, ZEBRA). The glomerulus-level classification model was exported in a format compatible with the WSInfer extension for QuPath^24^, enabling user-friendly, one-click inference on annotated glomerular regions, and uploaded in the following Github repository, where the technical functioning is also explained in the documentation:

https://github.com/Gizmopath/ZEBRA-ZEbra-Bodies-Recognition-by-Artificial-intelligence.

All code used during training and inference is included in the same repository, including the customized QuPath scripts for tile extraction and glomerular region annotation. The script for inference and quantification of foamy podocytes relative to total glomerular area is likewise available in the GitHub repository. However, unlike the glomerulus-level classification model, this script is provided as a standalone tool and is not directly compatible with integration into QuPath/WSInfer.

### A computational histology score for Fabry nephropathy: the ZEBRA score

**Fig. 4:**
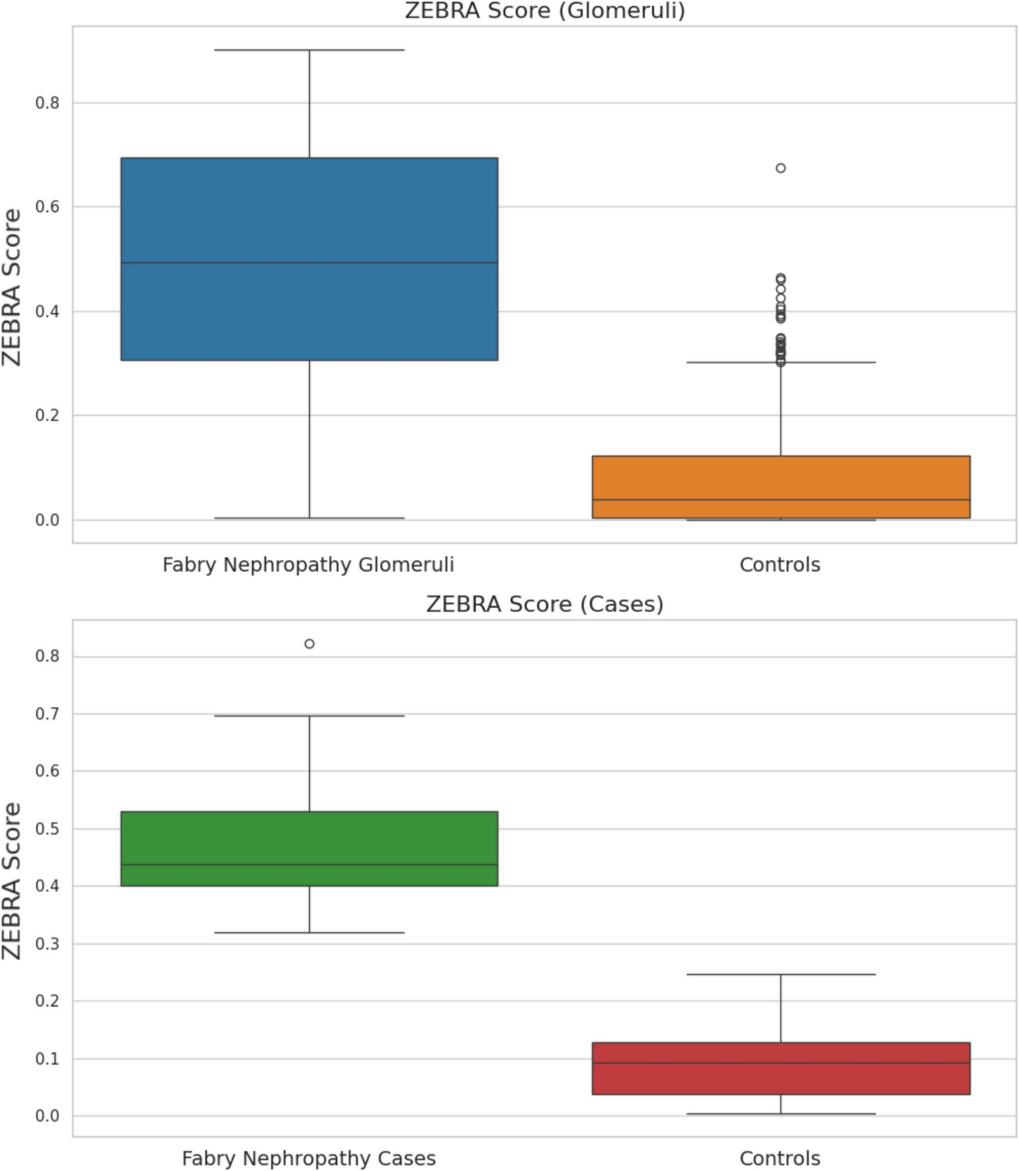
Comparative box plots of ZEBRA Scores between glomeruli from Fabry Nephropathy samples and glomeruli from samples with other renal diseases (top), and aggregated ZEBRA Scores per case (bottom). Statistically significant differences are observed in both comparisons, with a clear separation and no overlap at the case level.

**Fig. 5:**
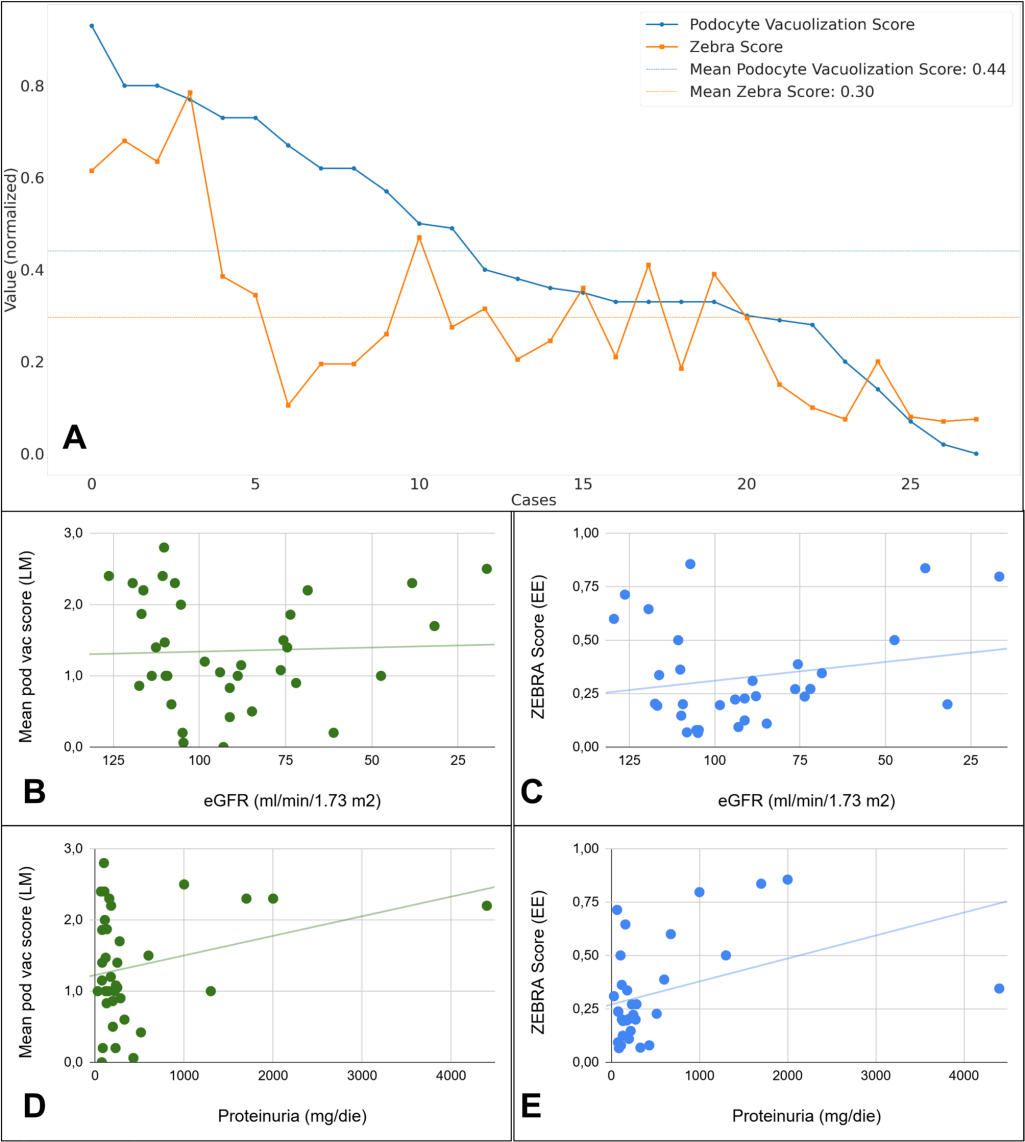
Comparison and correlations between the mean podocyte vacuolization score (MPVS), the ZEBRA Score (ZS), and clinical data. Panel (**A**) displays all positive cases arranged in descending order of their mean glomerular MPVS, alongside the corresponding ZS values for those same cases. Scatter plots with linear regression lines are shown for MPVS vs. eGFR (**B**), ZS vs. eGFR (**C**), MPVS vs. proteinuria (**D**), and ZS vs. proteinuria (**E**).

The obtained ZEBRA model was used to calculate the proportion of the area occupied by foamy podocytes (fpA) on the total glomerular area (tgA). The obtained average ZEBRA score (ZS = fpA/tgA %) further confirmed the capability of the algorithm to discriminate FNs from controls (Fig. 4), both at a case level (mean ZS of 0.49 ± 0.14 vs. 0.10 ± 0.8 in positive vs. negative cases, *p* < 0.001) and at a glomerular level (mean ZS of 0.5 ± 2.4 vs. 0.10 ± 0.13 in positive vs. negative cases, *p* < 0.001), with a clear separation and no overlap at the case level. ROC curve analysis of the ZEBRA score demonstrated good discrimination between FN and controls, with an AUC of 0.93. A preliminary cutoff of 0.19 (mean ZS per case) was identified, corresponding to a sensitivity of 0.91 and a specificity of 0.81. The comparison of ZS and MPVS for the positive cases demonstrated strong correlation between the two scores in both H&E (r_s_=0.66) and PAS (r_s_=0.71) stained slides, however, case-by-case analysis revealed substantial discrepancies between the two scores, likely due to the limited segmentation performance (Fig. 5A). Despite the subtle vacuolization potentially caused by X-chromosome inactivation in females, the model demonstrated non-inferior performance in females vs. males, with even higher correlation between MPVS and ZS in the former (rs = 0.74 for H&E, 0.75 for PAS) than in the latter group (rs = 0.56 for H&E, 0.68 for PAS). To assess the reliability and clinical relevance of the ZS even in cases with subtle clinical presentations, the analysis of this parameter in the subset of FN cases with eGFR > 90 mL/min/1.73 m² and proteinuria < 0.5 g/24 h showed a mean ZS of 0.22 ± 0.15, as compared to the null value of the control group with same characteristics, demonstrating the relevance even in this clinical subset. Linear regression analysis (Fig. 5B–E) indicated a modestly stronger correlation of ZS compared with MPVS with baseline clinical measures (eGFR-ZS R^2^ = 0.05 vs. 0.002 MPVS and proteinuria ZS R^2^ = 0.09 vs. 0.05 MPVS, respectively), although not reaching statistical significance (*p* = 0.88 and 0.26, respectively). Finally, to test the reliability of the algorithm we stressed the ZEBRA pipeline on a rare case of crystalline podocytopathy. Both the classification (EfficientNetB2) and the segmentation models (SegformerB4) detected positive glomeruli (5 out of 27) and/or podocytes (in 8 out of 27 glomeruli), respectively, but the final mean ZS was overall < 0.01, way below the proposed cutoff of 0.19, indicating a correct classification as non-FN case for this crystalline podocytopathy.

## Discussion

The integration of artificial intelligence (AI) into nephropathology has garnered significant attention, offering promising avenues for enhancing diagnostic precision and reproducibility in renal biopsy interpretation^20^. AI applications have been explored for tasks such as glomerular classification, segmentation, and quantification of histological features, aiming to augment traditional pathology workflow^25^. In the context of FN the diagnostic challenge is represented by the insidious clinical presentation that can be totally unspecific, especially in female patients and those with late-onset variants, which may reflect on relatively subtle modifications on renal biopsies (e.g. podocyte vacuolization) that can be overlooked by inexperienced pathologists thereby causing delays in treatment. Our study introduces a novel AI-driven pipeline, ZEBRA (Zebra bodies Evidenced on Bioptic Renal samples using Artificial intelligence), designed to automatically detect and quantify vacuolated podocytes in digitized renal biopsy slides to assist pathologists providing a first screening of H&E/PAS stained slides to highlight possible areas of glomerular accumulation of Gb3 within podocytes in the form of cytoplasmic vacuolization. Being quick and feasible, the developed tool may be applied at scale to routine biopsy series to uncover incidental FNs, which, based on our unpublished experience, may account for up to 1–2% of all renal biopsies. Moreover, it can be directly implemented on routine histology stains available in all the pathology labs, instead of needing epoxy-embedded toluidine blue-stained semi-thin section slides. Moreover, even if electron microscopy provides useful details that complement the light microscopy features of FN and recent reports demonstrate the applicability of AI algorithms on EM figures to assist renal pathologists in some evaluations (e.g. foot process effacement)^26,27^, we wanted to create an easily applicable tool to check widely available H&E/PAS slides with screening purpose to redirect the case to electron microscopy or genetic analysis.

The ZEBRA algorithm consists of two components: a glomerular-level classification module and a segmentation module that precisely delineates individual affected podocytes at the microscopic level. The glomerular detail of ZEBRA profits from the most recent and effective neural networks available for AI-based image processing and shows a perfect detection of all cases of FN in the test set while misdiagnosing some control glomeruli as affected, suggesting a good role for this tool as a screening assistant. This classification model has been built as a fully integratable extension for QuPath^28^ and WSInfer^24^ as a free resource to allow easy implementation for the daily work and prospective application of the tool to further validate its clinical utility. However, foamy podocytes are not exclusive to FD and may appear in other conditions, underscoring the need to interpret AI-based findings within the broader clinical and pathological context. Moreover, although the segmentation of ZEBRA achieved only a modest Dice score, reflecting the inherent complexity and variability of vacuolated podocyte patterns, it showed optimal tile-level sensitivity (0.95) and PPV (0.91) relying on state-of-the-art transformer models (SegFormerB4) and the careful annotations by expert renal pathologists as solid ground truth, providing the substrate for the elaboration of a computational disease burden quantification with the ZEBRA score. While the MPVS is useful to assess podocyte involvement, the manual assessment method can still have some limitations such as interobserver variability and inconsistent predictive capability. Our ZEBRA score aims to provide a reproducible and quantifiable measure of podocyte vacuolization, potentially serving as a biomarker for diagnostic and predictive purposes. While the correlations with baseline eGFR and 24-hour proteinuria were generally modest for both ZS and MPVS without statistically significant differences, a slight improvement was observed using ZS. In this experiment, the use of standard and widely available techniques, together with the implementation of key methodological strategies, including diverse data augmentation techniques and the use of multiple whole-slide scanner systems, enhanced the algorithm’s robustness and generalizability across a wide range of variability sources. These approaches directly address one of the major current challenges in translating AI algorithms into routine clinical practice: the risk of reduced performance when applied to external datasets^29^. Currently as a feasibility study due to limited sample size, the ZEBRA score correlates with baseline clinical parameters, whilst its predictive value concerning long-term outcomes remains to be elucidated. While controls were matched for center/time, they exhibited worse baseline renal function, which is a result of the retrospective selection of biopsy-proven non-Fabry glomerular diseases. Future prospective studies with larger cohorts and longitudinal follow-up are essential to validate the prognostic utility of the ZEBRA score, enhance its generalizability by expansion to multi-center external datasets with multi-annotator consensus, and its integration into clinical practice.

## Conclusion

This study introduces and validates a novel digital pathology pipeline for FN, culminating in the ZEBRA score, a new histological measure of podocyte involvement. The findings offer initial insights into its potential applications, emphasizing that the AI system provides a comprehensive analysis of image data, alerting human operators to possible oversights and helping prevent missed cases, in line with its design as a high-sensitivity screening tool. Future studies are warranted to assess the prognostic implications of the ZEBRA score and its utility in guiding therapeutic decisions in FD.

## Supplementary Information

Below is the link to the electronic supplementary material.

Supplementary Material 1

Supplementary Material 2

Supplementary Material 3

## Data Availability

Authors agree to make data and materials supporting the results or analyses presented in their paper available upon reasonable request. The trained models were exported in a format compatible with the WSInfer extension for QuPath, enabling user-friendly, one-click inference on annotated glomerular regions, and uploaded in the following Github repository: [https://github.com/Gizmopath/ZEBRA-ZEbra-Bodies-Recognition-by-Artificial-intelligence](https:/github.com/Gizmopath/ZEBRA-ZEbra-Bodies-Recognition-by-Artificial-intelligence).
