# Supplementary material for: Zebra bodies recognition by artificial intelligence (ZEBRA): a computational tool for Fabry nephropathy
Source: Sci Rep. 2026 Jan 12;16:5072. doi: 10.1038/s41598-026-35466-w (PMC12876834; doi:10.1038/s41598-026-35466-w)
Supplement: Supplementary file 1 — Supplementary Material 1 [file 41598_2026_35466_MOESM1_ESM.docx]

**Supplementary methods**

Images were split at the patient level into five stratified folds to ensure that all glomeruli or regions from a single subject were confined to either training or validation sets, thereby preventing data leakage. Data augmentation was applied during training to increase variability and prevent overfitting. Augmentations included random rotations (90°, 180°, 270°), horizontal and vertical flips, color jittering (modifications in brightness, contrast, saturation, hue), and Gaussian blurring. For segmentation tasks, these transformations were applied jointly to both images and masks to preserve spatial correspondence. Validation and test data underwent only resizing and tensor conversion. Input resizing was performed to match the input resolution requirements of each architecture. During training, class imbalance was handled by weighted random sampling based on inverse class frequency for classification and fold-specific foreground weights for the binary cross-entropy loss, computed from foreground-to-background pixel ratios in the training masks for segmentation.

All models were trained using the Adam optimizer (learning rate = 1×10⁻⁴) for a maximum of 40 epochs, with early stopping (patience = 5) based on validation performance. A ReduceLROnPlateau scheduler was used for segmentation models, with the Dice coefficient as the monitored metric.

Binary classification was performed to detect the presence of foamy podocytes within glomerular images. Multiple deep learning architectures were fine-tuned for this task. Model performance was assessed using accuracy, precision, recall, macro-averaged F1-score, and ROC-AUC, computed at each epoch. For each fold, the model achieving the highest validation F1-score was retained. The number of training epochs required to reach this performance was recorded, and the mean optimal epoch count across the five folds was used to train a final model on the entire training set. Final model performance was then evaluated on an independent test set using the same metrics.

Binary semantic segmentation was applied to localize glomerular regions and podocyte-associated lesions. Segmentation performance was monitored during training using the Dice coefficient and Intersection-over-Union (IoU), both computed on the training and validation sets. As with classification, model weights corresponding to the highest validation Dice score in each fold were retained. The average number of optimal epochs across folds was used to retrain a final model on the full training set, which was subsequently evaluated on a held-out test set.

The training, validation, and deployment procedures were performed using Python on a machine equipped with 64 GB of RAM and an NVIDIA GeForce RTX 4080 Super GPU.

**Supplementary results**

*Classification task (glomerular detail)*

The performance metrics of various deep learning architectures for the detection of foamy podocytes are summarized across training, validation, and test sets ([Table](#_v19w70m7m1x6) 3). Without data augmentation, all models showed strong performance during training (>92% accuracy), but experienced notable performance drops on the test set, particularly ResNet, EfficientNet, and DenseNet, indicating overfitting. Swin-T and UNI2 exhibited better generalization, with higher test F1 scores (55% and 65%, respectively) compared to other models. Data augmentation improved test performance across all architectures, with EfficientNet achieving the best overall test accuracy and F1 score (79%), followed by Swin-T (78%) and DenseNet (70%). These results suggest that transformer-based and hybrid models, especially when trained with augmentation, provide superior generalizability for this task.

*Segmentation task (podocyte detail)*

SegFormer outperformed both Unet and DeepLab in podocyte segmentation, achieving the highest test Dice score (46%) and IoU (37%), indicating superior spatial accuracy and consistency. However, all models showed limited absolute performances ([Table](#_ytfc07k1qo2b) 3). Data augmentation only modestly improved generalization in some cases (e.g., Unet test Dice increased from 16% to 26%). In contrast, glomerular segmentation using SegFormer achieved excellent performance, with test Dice and IoU scores of 94% and 88%, respectively, showing minimal variation across folds and no meaningful difference with augmentation.

**Supplementary Figure 1:** ROC curve analysis illustrates the discriminative performance of the ZEBRA score, with the area under the curve (AUC) reported and the optimal cutoff indicated.

**Supplementary Figure 2:** Examples of misclassified glomeruli in a rare crystalline podocytopathy case. In green, the segmented glomerular area and in orange the vacuolized podocytes are shown.
