# Supplementary figures and images for: Zebra bodies recognition by artificial intelligence (ZEBRA): a computational tool for Fabry nephropathy
Source: Sci Rep. 2026 Jan 12;16:5072. doi: 10.1038/s41598-026-35466-w (PMC12876834; doi:10.1038/s41598-026-35466-w)

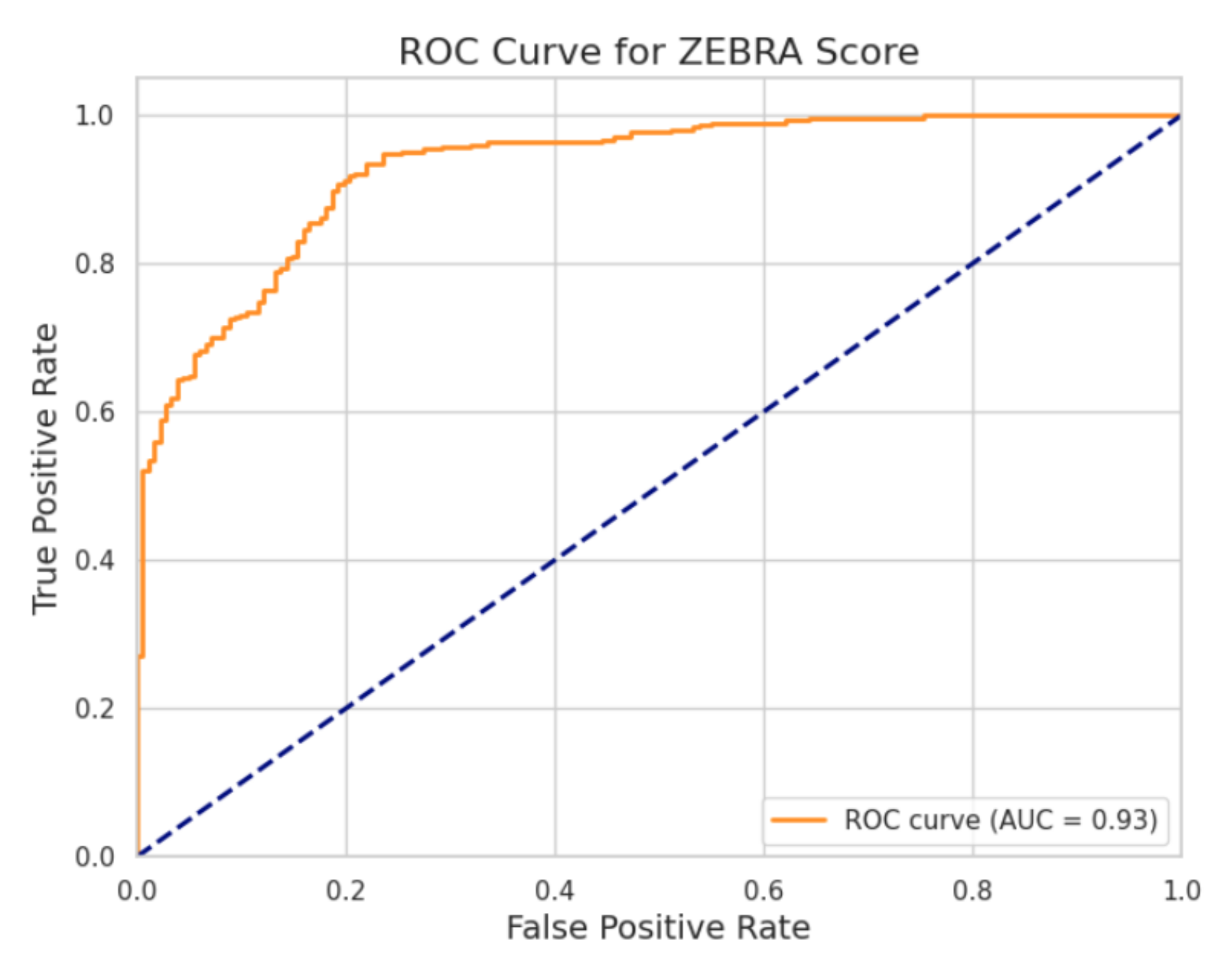

Supplement: Supplementary file 2 — Supplementary Material 2 [file 41598_2026_35466_MOESM2_ESM.tiff]

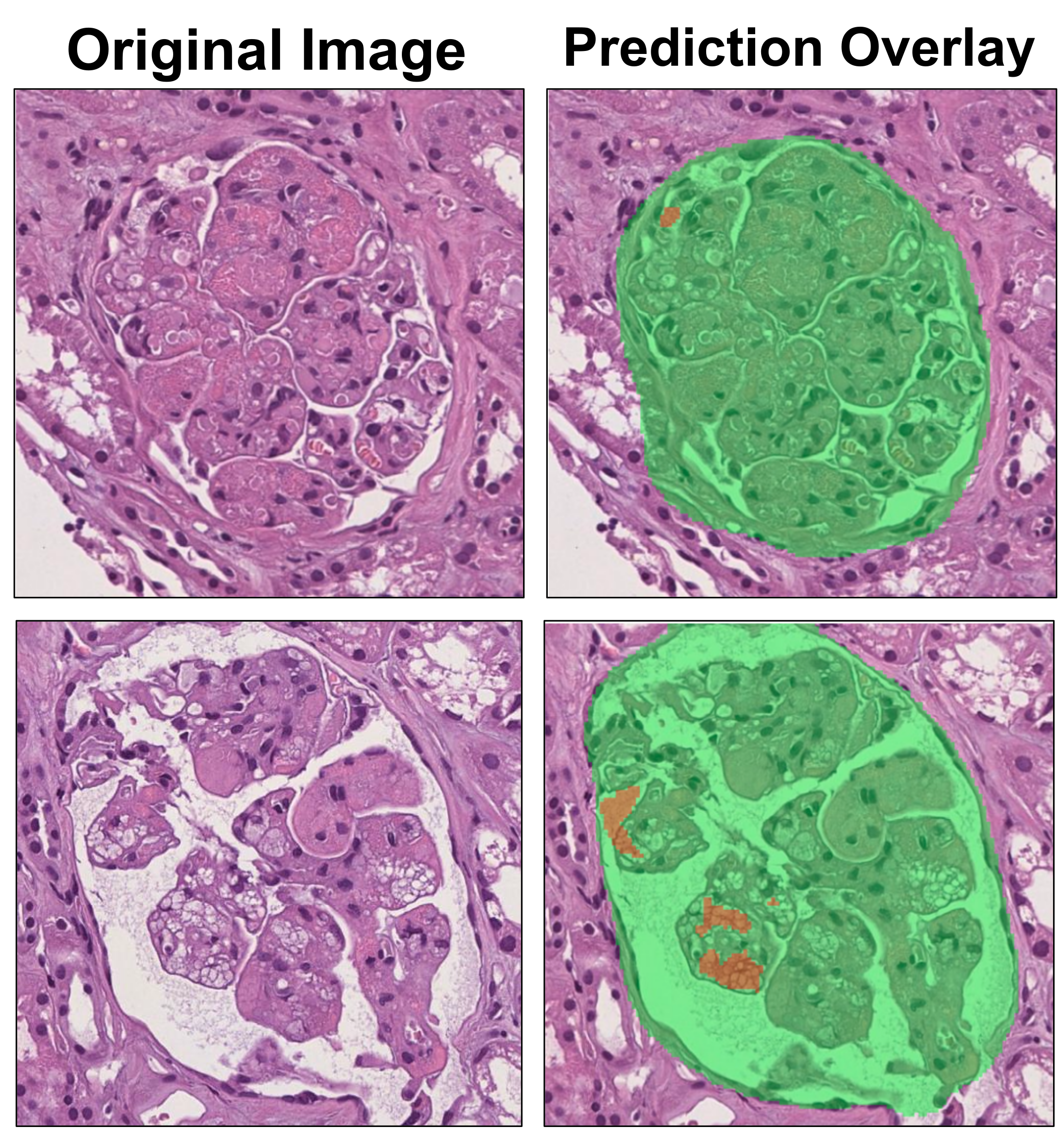

Supplement: Supplementary file 3 — Supplementary Material 3 [file 41598_2026_35466_MOESM3_ESM.tiff]
